# Regulation of *Gdf5* expression in joint remodelling, repair and osteoarthritis

**DOI:** 10.1038/s41598-019-57011-8

**Published:** 2020-01-13

**Authors:** Karolina Kania, Fabio Colella, Anna H. K. Riemen, Hui Wang, Kenneth A. Howard, Thomas Aigner, Francesco Dell’Accio, Terence D. Capellini, Anke J. Roelofs, Cosimo De Bari

**Affiliations:** 1 Arthritis and Regenerative Medicine Laboratory, Aberdeen Centre for Arthritis and Musculoskeletal Health, University of Aberdeen, Aberdeen, UK; 2 Interdisciplinary Nanoscience Center (iNANO), Department of Molecular Biology and Genetics, Aarhus University, Aarhus, Denmark; 3 Department of Pathology and Molecular Pathology, Medical Center Coburg, Coburg, Germany; 4 Centre for Experimental Medicine and Rheumatology, William Harvey Research Institute, Barts and the London School of Medicine and Dentistry, Queen Mary University of London, London, UK; 5 Department of Human Evolutionary Biology, Harvard University, Cambridge, Massachusetts USA; 6 Broad Institute of Harvard and MIT, Cambridge, Massachusetts USA

**Keywords:** Musculoskeletal models, Stem cells

## Abstract

Growth and Differentiation Factor 5 (*GDF5*) is a key risk locus for osteoarthritis (OA). However, little is known regarding regulation of *Gdf5* expression following joint tissue damage. Here, we employed *Gdf5-LacZ* reporter mouse lines to assess the spatiotemporal activity of *Gdf5* regulatory sequences in experimental OA following destabilisation of the medial meniscus (DMM) and after acute cartilage injury and repair. *Gdf5* expression was upregulated in articular cartilage post-DMM, and was increased in human OA cartilage as determined by immunohistochemistry and microarray analysis. *Gdf5* expression was also upregulated during cartilage repair in mice and was switched on in injured synovium in prospective areas of cartilage formation, where it inversely correlated with expression of the transcriptional co-factor Yes-associated protein (Yap). Indeed, overexpression of Yap suppressed *Gdf5* expression in chondroprogenitors *in vitro*. *Gdf5* expression in both mouse injury models required regulatory sequence downstream of *Gdf5* coding exons. Our findings suggest that *Gdf5* upregulation in articular cartilage and synovium is a generic response to knee injury that is dependent on downstream regulatory sequence and in progenitors is associated with chondrogenic specification. We propose a role for Gdf5 in tissue remodelling and repair after injury, which may partly underpin its association with OA risk.

## Introduction

Growth and Differentiation Factor 5 (*GDF5*) is a major risk locus for osteoarthritis (OA), the most common joint disease characterised by progressive loss of articular cartilage, remodelling of subchondral bone, chondro-osteophyte formation and synovitis. Common variants spanning a large 130 kb interval confer risk of hip and knee OA^1–3^. A well-studied SNP is located in the 5′ UTR of the *GDF5* gene (rs143383), with the OA susceptibility allele resulting in decreased *GDF5* expression^2,4–6^.

*Gdf5* plays important roles during joint formation. It is one of the earliest genes expressed in the embryonic joint interzone^7–10^, fated to give rise to joint tissues including articular cartilage, synovium, menisci, and ligaments^11,12^. *Gdf5*-expressing progenitors are continuously recruited into joint interzones throughout development^13^ and their progeny retain skeletal joint stem/progenitor activity in adulthood^14^. Following injury to the joint surface, *Gdf5*-lineage mesenchymal stromal/stem cells (MSCs) proliferate to underpin synovial hyperplasia and migrate to the site of injury, through the activity of the transcriptional co-factor Yes-associated protein (Yap), where they repair cartilage^14^.

Loss-of-function mutations in *GDF5* have been linked to congenital disorders including Hunter-Thompson syndrome^15^, brachydactyly type C^16^, and DuPan syndrome^17^. These syndromes are partly phenocopied in *brachypodism* (*bp*) mice, which harbour *Gdf5* coding mutations^7^. Homozygous *bp* mice have dysmorphic knees lacking cruciate ligaments^18,19^. Heterozygous *bp* mice, which model human *GDF5* variants that cause decreased *GDF5* expression, display no overt phenotype^19–21^ but show increased susceptibility to OA under experimental challenges^21^.

Recent studies using mice harbouring BAC transgenes have revealed a conserved *cis*-regulatory architecture for *GDF5* between humans and mice^19,22–24^. Regulatory sequences that control *Gdf5* expression in developing and adult joints are distributed over a hundred kilobases, including regions both upstream and downstream of its coding exons^22^. While *Gdf5* expression in the developing knee is driven by both upstream and downstream regulatory sequences, in adulthood downstream regulatory regions are uniquely used^19,22^, suggesting that the genomic sequences regulating continued expression of *Gdf5*/*GDF5* in the adult knee during homeostasis may be distinct. Of note, these downstream regions harbour a number of genetic risk variants for knee OA^3^.

In this study, we used BAC *Gdf5-LacZ* reporter mice^19,22^ to map *Gdf5* expression during adult knee joint tissue remodelling associated with OA development or acute cartilage injury and repair, and to determine whether a differential regulation of *Gdf5* expression is associated with such events.

## Methods

### Mice

All methods were carried out in accordance with relevant guidelines and regulations. All animal experimental protocols were approved by the UK Home Office and the Animal Welfare and Ethical Review Committee of the University of Aberdeen. Two *Gdf5* BAC transgenic mouse lines were used^19,22,23^. They both harbour a BAC transgene containing mouse *Gdf5* with an *IRES-LacZ* cassette in the 3’UTR. *Gdf5UP-LacZ* mice contain a modified BAC extending 110 kb upstream to 30 kb downstream of *Gdf5* coding exons, which includes a conserved regulatory region adjacent to the promoter upstream of the *Gdf5* coding exons. *Gdf5DOWN-LacZ* mice contain a modified BAC extending a further 109 kb downstream, which includes additional regulatory regions downstream of the *Gdf5* coding exons. Both lines were maintained as heterozygotes on an FVB background. *Gdf5-CreER* mice^13^ were provided by Dr. Elazar Zelzer (Weizmann Institute of Science, Israel) and crossed with Cre-inducible tdTomato (tdTom) reporter mice (Jackson Laboratory; B6.Cg-*Gt(ROSA)26Sor*^*tm14(CAG-tdTomato)Hze*^/J)^25^. Mice were group-housed in conventional cages on a 12:12 light-dark cycle, in a temperature-controlled room with water and food *ad libitum* and environmental enrichment provided. Tamoxifen (Sigma) dissolved in corn oil was administered by gavage at 6 weeks of age (180 mg/kg daily for 5 days), or to the pregnant dam at E11.5 (120 mg/kg), E13.5 (160 mg/ml) and E15.5 (160 mg/ml), and embryos were collected following euthanasia of the pregnant dam at E19.0.

### Surgical procedures

Male mice, 11–12 weeks old, underwent surgical unilateral destabilisation of the medial meniscus (DMM) on the left knee^26^ while the right knee served as internal control, and mice were euthanised 2 or 8 weeks later. Female mice, 9–11 weeks old, underwent surgery to induce unilateral joint surface injury by medial parapatellar arthrotomy as previously described^14^, and were euthanised 6–7 days or 4 weeks later. For all surgeries, isoflurane inhalation anaesthesia was used, and mice received a subcutaneous injection of 0.1 mg/kg Vetergesic (containing 0.3 mg/ml Buprenorphine) on the day of surgery and the following day. Mice were kept group-housed.

### X-gal staining

Whole-mount staining with X-gal to detect β-galactosidase (β-gal) activity was performed as described^27^, with modifications. Limbs were fixed in 4% PFA for 2 h at 4 °C, washed 3x in wash buffer (0.1 M phosphate buffer supplemented with 2 mM MgCl_2_, 0.01% sodium deoxycholate and 0.02% Igepal), stained with 0.75 mg/ml X-gal in staining solution (wash buffer supplemented with 4 mM potassium ferrocyanide, 4 mM potassium ferricyanide and 20 mM Tris buffer, pH 7.4) for 6 days at room temperature, then washed 3x in PBS. Limbs from wild-type mice were stained as controls.

### Human tissue collection

All human cartilage samples were obtained after informed consent and in accordance with the relevant guidelines and regulations, with approval from the NHS Grampian Biorepository Tissue Bank Committee. OA samples were obtained from five patients (47 to 79 years old, all female) undergoing knee arthroplasty. Normal samples were obtained from five joints (two knee joints, 1^st^ metatarsal phalangeal joint, ankle joint, talo-calcaneal joint) donated by three patients (40 to 59 years old, two males, one female) undergoing excision or amputation surgery for tumours unrelated to the joint sampled.

### Histology and immunohistochemistry

Samples were fixed in 4% PFA at 4 °C and decalcified in 10% EDTA in PBS. Samples were embedded and sectioned as described^14^. Sections were stained with Nuclear Fast Red (Vector Laboratories, UK) to stain nuclei, or with safranin-O (Sigma) to stain glycosaminoglycans in the cartilage matrix red, with fast green (Sigma) counterstain, following standard protocols. TRAP staining to detect osteoclasts was carried out using a TRAP staining kit (Sigma). Immunohistochemistry was performed as described^28,29^ using antibodies listed in Supplementary Table 1. Collagen type II was detected following enzyme-based antigen retrieval with 1.5 mg/ml porcine pepsin (Sigma) for 45 min at 37 °C. Yap and GDF5 were detected following antigen retrieval for 4 hours at 80 °C in antigen unmasking citrate buffer solution (pH 6, Vector Laboratories, UK). Stained sections were imaged using a Zeiss Axioscan Z1 slide scanner (Carl Zeiss Ltd, UK), Zeiss Axioskop 40 (Zeiss) with Progress XT Core 5 colour digital camera and ProgRes CapturePro 2.9.0.1 software (JenOptik, Germany), or 710 META Laser-Scanning Confocal Microscope with ZEN software (Zeiss) and analysed using ZEN2 (blue edition, Carl Zeiss Ltd). Cartilage damage of the tibial plateau was assessed using the Osteoarthritis Research Society International (OARSI) scoring system^30^.

### Quantification of X-gal staining

Colour deconvolution was applied to images of X-gal-stained sections to remove the Nuclear Fast Red counterstaining using ImageJ with Fiji package and Colour Deconvolution Plugin (Dr. Gabriel Landini, University of Birmingham, UK) based on published methods^31^. All images were acquired with the same magnification, resolution and light settings. The number, size and staining intensity of X-gal-stained chondrocytes in the tibial cartilage was then determined by creating a binary image using thresholding and watershedding, and analysing particles by redirecting measurements to matching greyscale images. Four sections per sample were analysed. Total X-gal staining was calculated by multiplying the number and staining intensity of X-gal-stained chondrocytes.

### Primary cell isolation and *in vitro* chondrogenesis

Cells were isolated from *Gdf5* BAC mouse knees as described^14^. Chondrogenesis was induced in high-cell density pellet culture (2.5–3 × 10^5^ cells) with 10 ng/ml TGFβ1 (Gibco) or 300 ng/ml BMP-2 (Prospec) for 21 days, as described^14^. Pellets were fixed in 4% PFA for 15 min, X-gal-stained for 4 h and post-fixed for 15 min, cryoprocessed, sectioned and stained with Toluidine Blue or Nuclear Fast Red.

### Overexpression and knockdown experiments

C3H10T1/2 cells (American Type Culture Collection, USA) were retrovirally transduced to express wildtype or constitutively active YAP1, as described^32^. Cells were seeded in monolayer (15,000/cm^2^), transduced the next day, and RNA extracted 2 days later. Alternatively, transduced cells were seeded in high-cell density micromass culture (4 × 10^5^ cells) in chemically-defined serum-free medium (high-glucose DMEM with glutamine, supplemented with 50 μg/ml ascorbic acid, 1 mg/ml recombinant human insulin, 0.55 mg/ml transferrin, 0.5 ug/ml sodium selenite, 50 mg/ml BSA and 470 µg/ml linoleic acid)^32^, and the next day RNA was extracted. For knockdown experiments, cells were seeded at 42,000/cm^2^ and transfected the next day with DsiRNA (Supplementary Table 2) (Integrated DNA Technologies, USA) using Mirus TransIT-X2 reagent (Mirus Bio LLC, USA). The following day, cells were seeded in micromass culture (2.5–3 × 10^5^ cells) and cultured under chondrogenic conditions by treatment with 300 ng/ml BMP-2, as described^32^. After 4 days, RNA was extracted for analysis of gene expression.

### Gene expression analysis

Total RNA was extracted using TRIzol reagent (Invitrogen, Paisley, UK) according to standard protocols, and RNA was quantified using a NanoDrop ND-1000 spectrophotometer (Labtech, Uckfield, UK). cDNA was synthesised using random hexamer primers and SuperScript Reverse Transcriptase (Invitrogen), according to manufacturer’s instructions. Quantitative PCR (qPCR) was performed with a Roche LightCycler 480 using SYBR Green Master (Roche), according to standard protocols. Expression of genes of interest was normalised to expression of *Hprt1*. Primer sequences are listed in Supplementary Table 3.

### Statistical analysis

Microarray data were analysed using Bioconductor (Affy package for pre-processing and normalization and Limma for statistical comparison of expression levels using a false-discovery-rate of 5%). Principal component analysis was performed using the prcomp package in R. All other data were analysed using GraphPad Prism v5 and SigmaPlot v13. A p-value ≤0.05 was considered statistically significant. For comparison of two groups, two-tailed t-test was used. For comparison of ≥3 groups, one-way or two-way ANOVA with Holm-Sidak post-test was used. Data following a lognormal distribution were log-transformed for statistical testing. N-numbers and data points on graphs represent individual mice, patients, or *in vitro* experiments, with horizontal lines indicating mean.

## Results

### *Gdf5* expression in OA

To investigate *Gdf5* expression in experimentally induced OA, we used two *Gdf5*-*LacZ* reporter mouse lines^22^. *Gdf5UP-LacZ* mice contain a BAC extending 110 kb upstream to 30 kb downstream of *Gdf5* coding exons, which includes a conserved regulatory region adjacent to the promoter upstream of the *Gdf5* coding exons. *Gdf5DOWN-LacZ* mice contain a BAC extending a further 109 kb downstream, which includes additional regulatory regions downstream of the *Gdf5* coding exons that are not present in the *Gdf5UP-LacZ* BAC. Both BACs were modified to contain an *IRES-LacZ* cassette in the 3′UTR of the *Gdf5* gene, thus *LacZ* expression is indicative of the activity of the *Gdf5* regulatory regions contained within the BAC^22^. While both mouse lines express *LacZ* in the knee during development^19,22^, only *Gdf5DOWN-LacZ* mice express *LacZ* in the knee in adulthood (Supplementary Fig. 1)^19^. The *Gdf5DOWN-LacZ* BAC is also able to rescue the knee phenotype in *bp* mice, indicating it contains the regulatory regions necessary for adequate expression in the knee^19^. Here, we found that the *LacZ* expression pattern in *Gdf5DOWN-LacZ* adult knees resembled the tdTom labelling pattern in knees from adult mice with a knock-in of *CreER* at the endogenous *Gdf5* locus^13^ crossed with Cre-inducible *tdTom* reporter mice^25^ shortly after tamoxifen induction (Supplementary Fig. 2A,B). TdTom labelling was sparse, likely due to inefficient Cre-recombination as observed in embryos (Supplementary Fig. 2C–E)^13^. Nonetheless, these data support *LacZ* expression in knees from adult *Gdf5DOWN-LacZ* mice as reflecting transcriptional activity of endogenous *Gdf5*.

**Figure 1:**
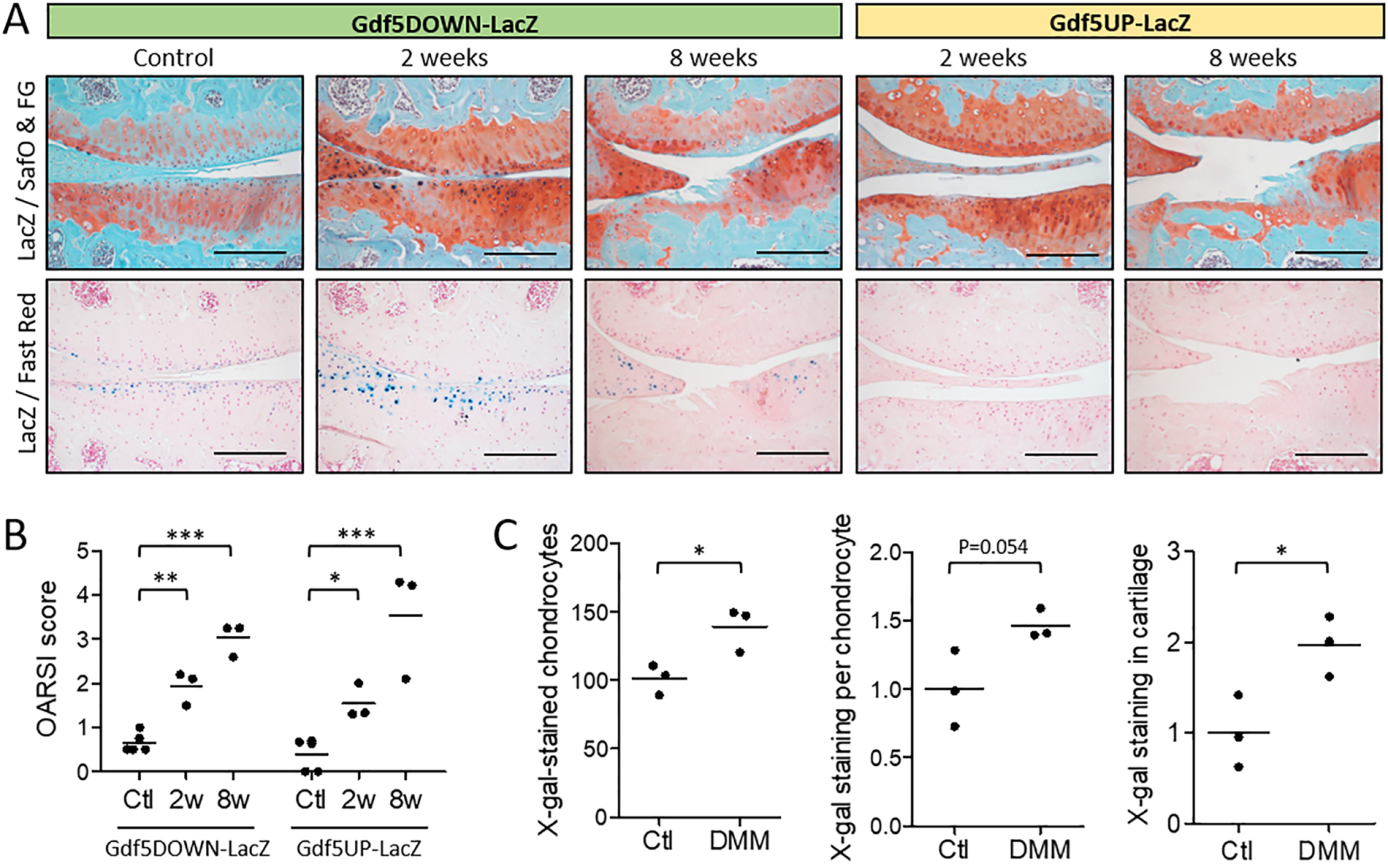
*Gdf5* expression in articular cartilage following DMM. (**A**) *LacZ* expression (blue X-gal staining) in the articular cartilage of the medial femoral condyle (top of images) and tibial plateau (bottom of images) at 2 and 8 weeks after DMM, or in contralateral control knee, in *Gdf5DOWN-LacZ* and *Gdf5UP-LacZ* mice (n = 3 for both strains and both timepoints). At 2 weeks, cartilage shows focal loss of proteoglycan staining (red Safranin O staining) and minor fibrillations at the surface, while at 8 weeks it is severely damaged. LacZ, whole-mount X-gal staining to detect *LacZ* expression; SafO & FG, Safranin O and Fast Green counterstaining; Fast Red, Nuclear Fast Red counterstaining. Scale bars, 200 μm. (**B**) OARSI histopathological scores of cartilage damage of the medial tibial plateau at 2 weeks (2w, n = 3) or 8 weeks (8w, n = 3) after DMM surgery, or no surgery (Ctl, n = 5). *p < 0.05; **p < 0.01; ***p < 0.001, two-way ANOVA with Holm-Sidak post-test for comparisons against control. There were no significant differences between the two mouse lines. (**C**) Number of counted X-gal-stained chondrocytes, average X-gal staining intensity per chondrocyte, and X-gal staining in cartilage calculated by multiplying number and staining intensity of X-gal-stained chondrocytes, in tibial articular cartilage of *Gdf5DOWN-LacZ* mice at 2 weeks after DMM. Data are expressed relative to the internal contralateral control knees (Ctl). *p < 0.05, two-tailed Student’s t-test.

We analysed *LacZ* expression in the knees of *Gdf5-LacZ* mice after DMM (Fig. 1A,B). In *Gdf5DOWN-LacZ* mice, increased *LacZ* expression was observed in medial compartment articular cartilage at 2 weeks, particularly in areas with early signs of damage, as shown by loss of Safranin O staining which stains proteoglycans in the cartilage extracellular matrix (Fig. 1A). Quantification showed an increase in both the number of *LacZ*-expressing chondrocytes and average X-gal staining intensity per chondrocyte (Fig. 1C), resulting in a significantly higher overall *LacZ*-expression in the medial tibial plateau cartilage in DMM knees. At 8 weeks after DMM, *LacZ* expression persisted in articular cartilage of *Gdf5DOWN-LacZ* mice but was less pronounced and undetectable in areas of severe damage (Fig. 1A). In *Gdf5UP-LacZ* mice, no *LacZ* expression was detectable in the cartilage at either time-point (Fig. 1A). These data indicate that *Gdf5* downstream regulatory elements are activated in articular chondrocytes in the early phase of OA.

**Figure 2:**
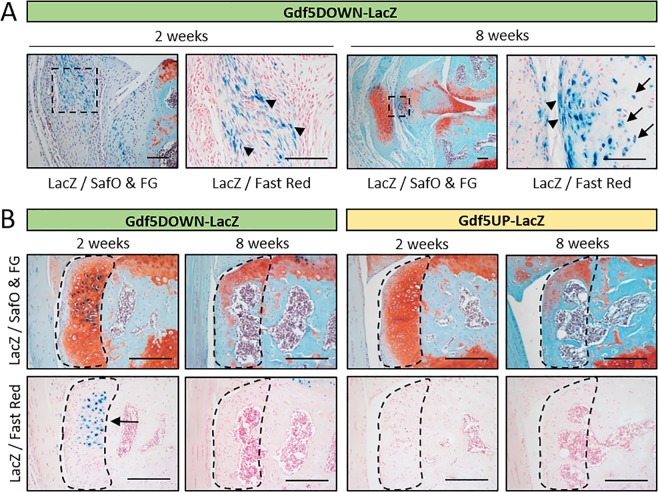
*Gdf5* expression during ectopic cartilage formation *in vivo*. (**A**) *LacZ* expression in fibroblast-like cells (blue, arrowheads) in medial synovium at 2 and 8 weeks post-DMM in *Gdf5DOWN-LacZ* mice (n = 3). At 8 weeks post-DMM, ectopic cartilage in synovium was observed with *LacZ*-expressing chondrocytes (blue, arrows) and surrounding *LacZ*-expressing fibroblast-like cells (blue, arrowheads). (**B**) Chondrophytes at 2 weeks post-DMM and mature osteophytes at 8 weeks post-DMM (indicated by dashed lines) showing *LacZ*-expressing chondrocytes (blue) in the chondrophytes in *Gdf5DOWN-LacZ* mice, but not *Gdf5UP-LacZ* mice, at 2 weeks post-DMM (n = 3 for both strains and both time points). LacZ, whole-mount X-gal staining to detect *LacZ* expression; SafO & FG, Safranin O and Fast Green counterstaining; Fast Red, Nuclear Fast Red counterstaining. Scale bars, (**A**) 100 μm, (**B**) 200 μm.

*LacZ* expression was also detected in the medial synovium of *Gdf5DOWN-LacZ* mice at 2 weeks post-DMM and remained detectable at 8 weeks, specifically in ectopic chondrocytes and surrounding fibroblast-like cells (Fig. 2A). In addition, *LacZ* was expressed in chondrophytes at 2 weeks post-DMM but was no longer detectable in mature osteophytes at 8 weeks (Fig. 2B). *LacZ* expression was not detected in knees from *Gdf5UP-LacZ* mice at either time-point (Fig. 2B). We infer that *Gdf5* is expressed in areas of forming ectopic cartilage during OA.

**Figure 3:**
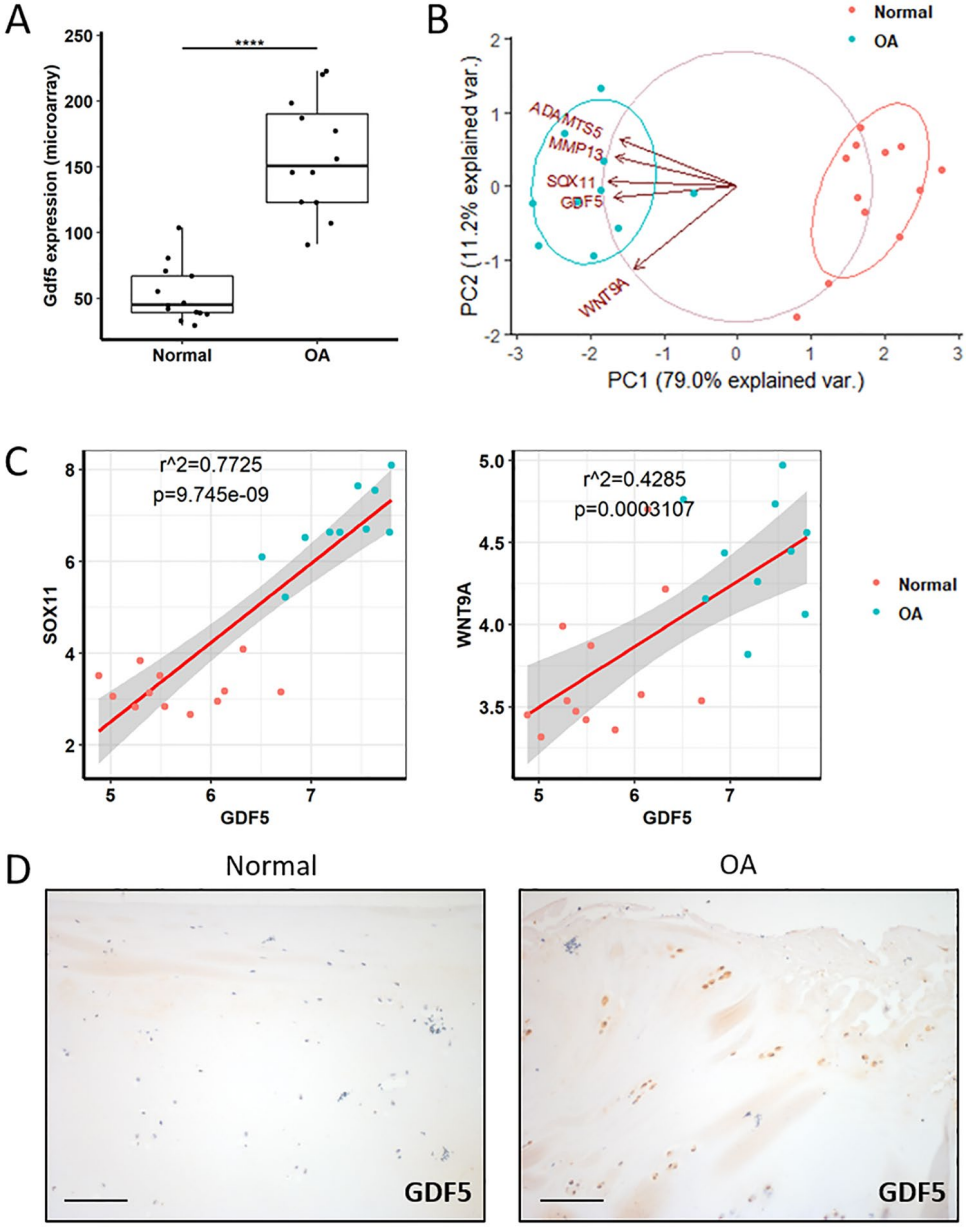
Expression of GDF5 and upstream regulators in human OA cartilage. Gene expression data were obtained by mining a previously published microarray comparing normal versus OA human knee cartilage^33^. (**A**) *Gdf5* expression was higher in OA cartilage compared to normal cartilage. Linear modelling (limma) with Benjamini-Hochberg correction for multiple comparisons. ****p < 0.0001. (**B**) Principal component analysis including the genes indicated showed complete separation of the normal samples from the OA samples. PCA was performed with the prcomp function in R. (C) Linear regression modelling showed *GDF5* expression to strongly correlate with the expression of *SOX11* (left) and *WNT9a* (right). (D) IHC staining for GDF5 in articular cartilage samples from patients with OA (n = 5 donors) in comparison to normal cartilage (n = 3 donors; 5 joints). Scale bars, 100 μm. See also Supplementary Fig. 3.

For clinical relevance, we analysed data from published microarrays of human cartilage from knees of normal donors and OA patients^33^. *GDF5* expression was upregulated in the cartilage of OA patients (Fig. 3A), alongside increased expression of cartilage degrading proteins known to be upregulated in OA (*MMP13*, *ADAMTS5*) (Fig. 3B). *GDF5* expression correlated with expression of *SOX11* and *WNT9A* (Fig. 3B,C), known upstream regulators of *Gdf5* expression during development^34–36^, indicating these factors may also modulate *GDF5* expression in human articular cartilage during OA. Immunohistochemistry for GDF5 on articular cartilage samples from a distinct cohort of OA patients and controls confirmed GDF5 was upregulated in OA cartilage (Fig. 3D and Supplementary Fig. 3).

### *Gdf5* expression following joint surface injury

**Figure 4:**
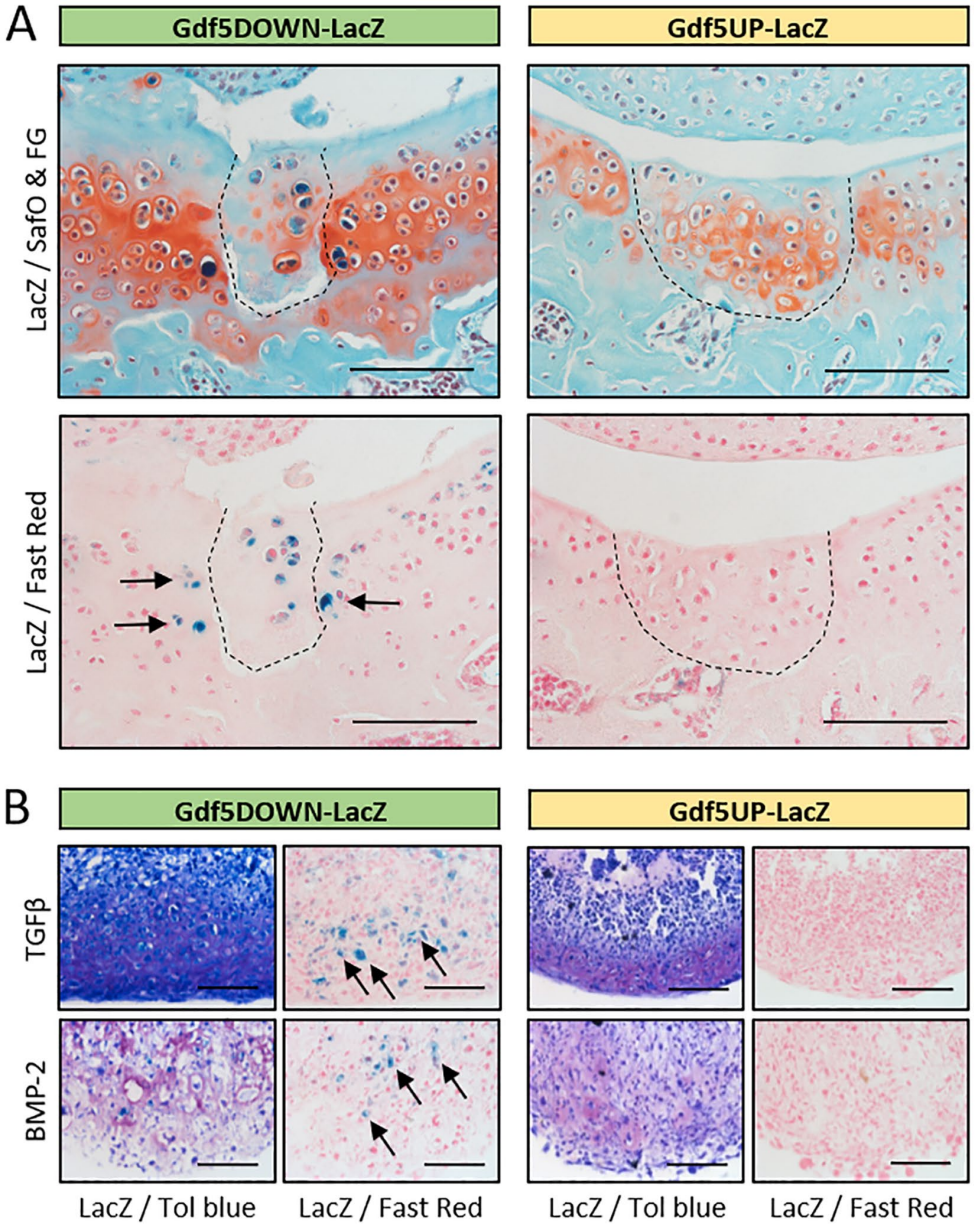
*LacZ* expression is upregulated during cartilage repair and *in vitro* chondrogenesis. (**A**) Areas of healed cartilage (dashed line) in the patellar groove of the femur of *Gdf5DOWN-LacZ* (n = 4/10) and *Gdf5UP-LacZ* (n = 3/10) mice, with *LacZ*-expressing chondrocytes (blue, arrows) detected in *Gdf5DOWN-LacZ* mice 4 weeks post-injury. LacZ, whole-mount X-gal staining to detect *LacZ* expression; SafO & FG, Safranin O and Fast Green counterstaining; Fast Red, Nuclear Fast Red counterstaining. Scale bars, 100 μm. (**B**) Histological sections of chondrogenic cell pellets. Synovial cells were isolated from *Gdf5DOWN-LacZ* and *Gdf5UP-LacZ* mice and treated *in vitro* for 21 days with TGFβ (10 ng/ml) or BMP-2 (300 ng/ml) to induce chondrogenesis, followed by X-gal staining to detect *LacZ* expression. Tol blue, Toluidine blue metachromatic staining indicates deposition of cartilage proteoglycans; Fast Red, Nuclear Fast Red counterstaining. *LacZ*-expressing chondrocytes (blue, arrows) were observed in *Gdf5DOWN-LacZ* cell pellets, but not *Gdf5UP-LacZ* pellets, under both culture conditions. Scale bars, 100 μm.

To investigate *Gdf5* expression during cartilage repair, we analysed *LacZ* expression in the *Gdf5-LacZ* transgenic mice 4 weeks after joint surface injury. In *Gdf5DOWN-LacZ* mice, chondrocytes in the repair tissue strongly expressed *LacZ*. We also detected prominent *LacZ* expression in chondrocytes in the native cartilage immediately adjacent to the repair tissue (Fig. 4A). In contrast, no staining was observed in repaired cartilage in *Gdf5UP-LacZ* mice (Fig. 4A). In support of these findings, while undetectable in monolayer culture, *LacZ* expression was detected in MSCs isolated from the knees of *Gdf5DOWN-LacZ* mice following chondrogenic differentiation in pellet culture, but not in chondrogenic pellets of *Gdf5UP-LacZ* MSCs (Fig. 4B). These data indicate upregulation of *Gdf5* expression, mediated by downstream regulatory regions, during articular cartilage repair.

**Figure 5:**
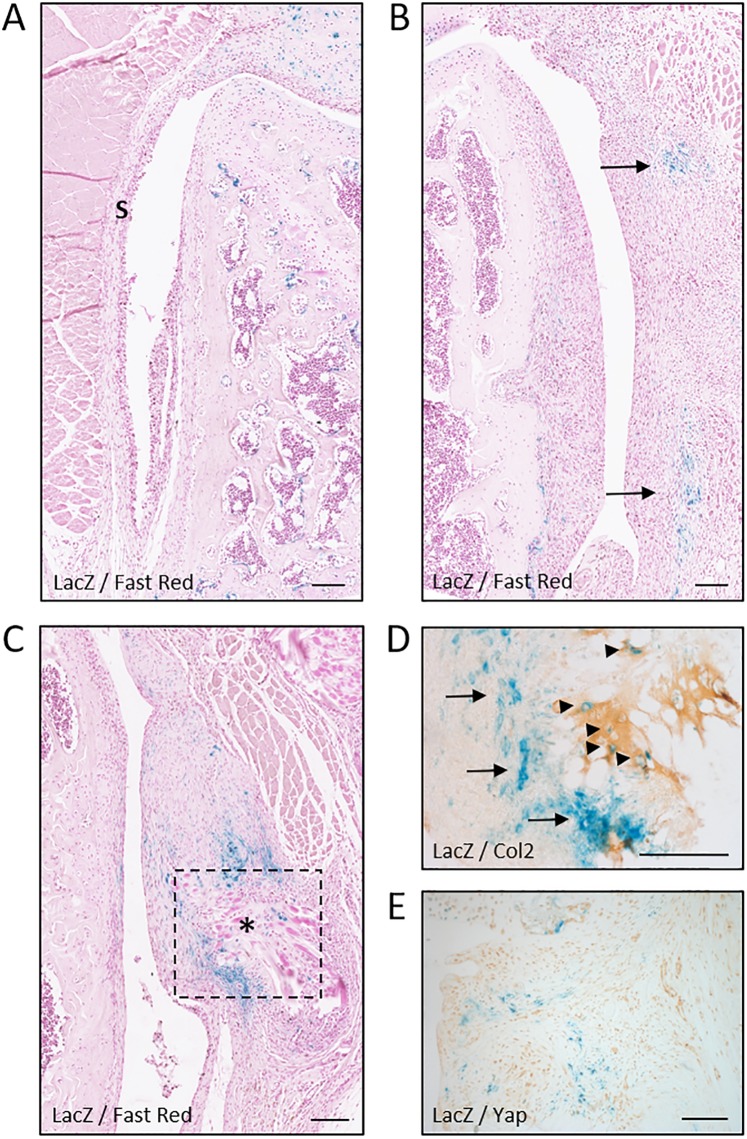
*Gdf5* is switched on in areas undergoing ectopic cartilage formation in synovium. *LacZ* expression in lateral (**A**) and medial synovium (**B**–**E**) from *Gdf5DOWN-LacZ* mice 1 week (**A**,**B**) or 4 weeks (**C**–**E**) after joint surface injury (n = 4 for both timepoints). (**A**) *LacZ* expression was not detected in synovium (S) on the lateral side. (**B**) Clusters of *LacZ*-expressing fibroblast-like cells (blue, arrows) were found in the medial synovium, near the site of surgical incision. (**C**) *LacZ* expression in medial synovium persisted at 4 weeks after injury, particularly near surgical sutures (asterisk). Dotted line indicates area shown in (**D**) in a consecutive section. (**D**) IHC staining for Collagen type II (Col2; light brown) revealing *LacZ*-expressing chondrocytes (blue, arrowheads) embedded in a cartilage matrix surrounded by *LacZ*-expressing fibroblast-like cells (blue, arrows). (**E**) IHC staining for Yap showing *LacZ*-expressing cells (blue) with little or no Yap interspersed between Yap-expressing cells (light brown) that did not detectably express *LacZ*. LacZ, whole-mount X-gal staining to detect *LacZ* expression; Fast Red, Nuclear Fast Red counterstaining. Scale bars, 100 μm.

Since *LacZ* was switched on in *Gdf5DOWN-LacZ* MSCs during chondrogenesis, we next analysed the synovium, which contains stem/progenitor cells that can undergo chondrogenic differentiation following injury and are postulated to repair injured cartilage^14,28,37^. *LacZ* was not detectable in synovium during homeostasis in either model (Supplementary Fig. 1). One week after joint surface injury, the synovium was hyperplastic, as expected^28,38^. In the synovium on the lateral side of the knee, not incised during surgery, *LacZ* remained undetectable in both mouse lines at both time-points (Fig. 5A and not shown), indicating that *Gdf5* expression is not switched on in synovium in response to cartilage injury. However, in synovium on the medial side, which was incised during surgery, small clusters of *LacZ*-expressing cells with a fibroblast-like morphology were detected in *Gdf5DOWN-LacZ* mice (Fig. 5B), and such cells persisted at 4 weeks after injury (Fig. 5C). They were predominantly localized near surgical sutures, where fibroblast-like cells that stained strongly for β-gal were observed around small clusters of *LacZ*-expressing chondrocytes embedded in a matrix containing collagen type II (Fig. 5D). Thus, as in DMM mice, *Gdf5* expression is upregulated in synovium in areas of prospective cartilage formation, suggesting a role for Gdf5 in chondrogenic specification and differentiation.

### Yap suppresses *Gdf5* expression in chondroprogenitors

**Figure 6:**
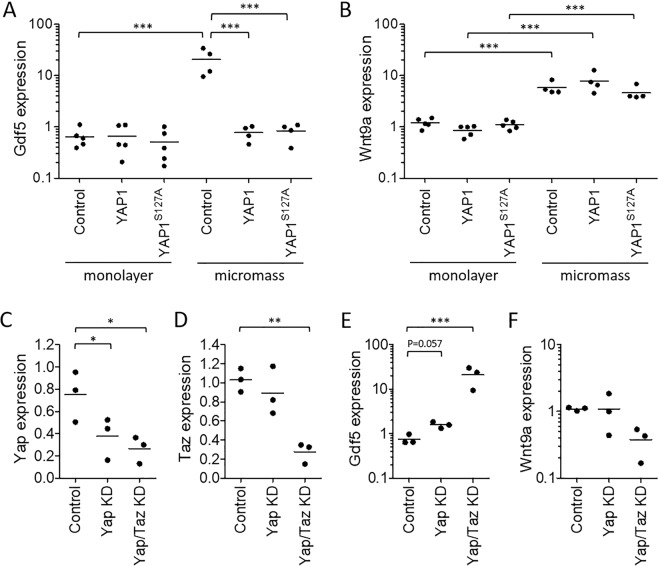
Yap supresses *Gdf5* expression. (**A**,**B**) C3H10T1/2 cells were transduced with retrovirus encoding YAP1 or constitutively active YAP1^S127A^, or with empty vector (Control), and cultured in standard monolayer (n = 5 experiments), or in high-density micromass for 1 day (n = 4 experiments; data from separate experiments). (**C**–**F**) C3H10T1/2 cells were transfected with DsiRNA to knock down Yap, or Yap and Taz, or with mismatch DsiRNA (Control), and cultured for 4 days in chondrogenic micromass culture. *Gdf5* (A,E), *Wnt9A* (**B**,**F**), *Yap* (**C**) and *Taz* (**D**) expression were determined by quantitative RT-PCR. All data were normalised to expression of *Hprt1*, and are shown relative to expression of the gene of interest in non-transduced cells in monolayer (**A**,**B**), or in micromass (**C**–**F**). *p < 0.05; **p < 0.01; ***p < 0.001, based on two-way ANOVA with Holm-Sidak post-test for pairwise comparisons (**A**,**B**) or one-way ANOVA with Holm-Sidak post-test for comparisons against control (**C**–**E**).

We previously reported that Yap is upregulated in synovium after joint surface injury and is required for the local expansion of *Gdf5*-lineage MSCs and their recruitment to the cartilage defect^14^, whereas Yap prevents chondrogenic differentiation^32^. Here, we compared expression of *LacZ* and Yap in *Gdf5DOWN-LacZ* mouse knees after joint surface injury and observed areas in synovium where Yap and *LacZ* showed an inverse expression pattern, with cells that expressed *LacZ* showing diminished Yap compared to surrounding cells (Fig. 5E). We hypothesized that high Yap activity during cell proliferation inhibits chondrogenic differentiation, as reported^32^, by actively suppressing chondrogenic factors including *Gdf5*. Hence, we determined the effect of overexpression of Yap on *Gdf5* expression in high-cell-density cultures using murine C3H10T1/2 MSCs. After one day of high-cell-density micromass culture, *Gdf5* expression was upregulated approximately 20-fold when compared to cells in monolayer (Fig. 6A), as previously reported with human synovial MSCs^39^. Strikingly, overexpression of YAP1 prevented the upregulation of *Gdf5* in micromass (Fig. 6A). In contrast, YAP1 overexpression failed to prevent the upregulation of *Wnt9a*, known to be upstream of *Gdf5*^35^, even when cells were transduced to express constitutively active YAP1^S127A^ (Fig. 6B). Conversely, knockdown of Yap in C3H10T1/2 MSCs in micromass increased *Gdf5* expression, an effect that was synergistically enhanced with concomitant knockdown of the paralog of Yap, Transcriptional Co-Activator with PDZ binding motif (Taz) (Fig. 6C–E). *Wnt9a* expression was not similarly modulated by Yap and Taz knockdown (Fig. 6F). Altogether, these data identify Yap as a negative regulator of *Gdf5* expression in chondrogenic MSCs, and indicate that Yap acts downstream of *Wnt9a*, possibly by directly modulating the activity of one or more transcription factors acting on *Gdf5 cis*-regulatory elements.

## Discussion

Allelic variants at the *GDF5* locus have been linked to OA risk, suggesting GDF5 plays important roles in joint maintenance throughout life. Expression of Gdf5 in adult articular cartilage has been reported in mice^40^ and humans^5,41^, with upregulation in OA^41^. Little was known regarding *Gdf5* expression in response to acute joint surface defects, which can progress to OA in the absence of repair^42^, or during the different stages of OA. Here, we show *Gdf5* expression in remodelling joint tissues, using two BAC *LacZ* reporter mouse strains harbouring distinct yet partially overlapping regions of the *Gdf5* locus^19,22^. After joint surface injury, *Gdf5* was highly expressed in chondrocytes both inside the newly formed cartilage repair tissue and in the adjacent stressed cartilage. Similarly, *Gdf5* was upregulated in cartilage during early-stage OA, particularly in areas of initial damage, and was detected in forming chondrophytes. Given the known chondrogenic activity of Gdf5^12,43^ our findings implicate a role for Gdf5 in new cartilage formation following injurious events in adulthood, possibly representing an attempt to repair joint damage. During late-stage OA, areas of advanced cartilage damage displayed markedly reduced LacZ staining, in line with previous studies reporting decreased *Gdf5* expression in extensively damaged cartilage in mice with inflammatory or degenerative arthritis^34,40^. These data support a role for Gdf5 in the maintenance and repair of articular cartilage in adult life, and provide a rationale for the administration of exogenous Gdf5 to aid cartilage repair in OA treatment^44^.

We show that *Gdf5* expression after injury and during OA is dependent on DNA sequence more than 30 kb downstream from the *Gdf5* coding region. This downstream sequence contains joint-specific regulatory elements^22^, and is both capable of, and necessary for, rescuing the *bp* knee phenotype in mice^19,22–24^. Importantly, it harbours many common risk variants for OA, of which several reside in known enhancers. Our findings indicate that such downstream variants may confer OA risk partly through modulating *Gdf5* expression in the adult knee in response to injurious events, thereby impacting on joint maintenance and reparative processes. They further indicate that the effect of a human variant such as the rs143383 SNP in the 5′UTR^2,4–6^ is likely to be dependent on *cis-*acting variants present in downstream *cis*-regulatory elements that are critical to drive adequate expression of *Gdf5*. Whether downstream regulatory elements involved in repair are different from those involved in OA development remains to be determined.

The identification of molecules that regulate *Gdf5* expression will provide critical insights into joint formation, maintenance and disease. We have unveiled a regulatory mechanism, to our knowledge hitherto unreported, that links Yap activity to *Gdf5* expression. Undetectable in quiescent synovium, *Gdf5* was switched on in activated chondroprogenitors in synovium following injury, concomitant with Yap downregulation. In chondrogenic MSCs, Yap suppressed expression of *Gdf5* but not *Wnt9a*, known to induce *Gdf5* expression^35,36^. Our data indicate that Yap negatively regulates *Gdf5* expression, possibly downstream of Wnt9a, and we propose that Yap needs to be down-regulated to enable *Gdf5* expression to prime progenitors towards chondrogenesis. Indeed, Yap prevents MSC chondrogenic differentiation *in vitro*^32^. Candidate transcription factors that could partner with Yap to regulate *Gdf5* include Sox11, reported to directly regulate *Gdf5* expression^34^ and found here to correlate with *GDF5* expression in human OA cartilage, and ZEB1, since ZEB1 binding sites are present in the enhancer upstream of the *Gdf5* promoter region^22^ and a direct interaction between ZEB1 and Yap has been reported^45^.

In conclusion, Gdf5 is upregulated in stressed cartilage, switched on in chondroprogenitors and expressed in newly forming cartilage during tissue remodelling following knee injury. This is dependent on activity of downstream regulatory sequence and occurs irrespective of whether the injury is acute or the result of chronic joint instability, indicating that Gdf5 modulation is not linked to a specific injurious event. An understanding of the regulation of Gdf5 in the context of remodelling, repair and OA pathogenesis will have important implications for joint surface regenerative therapies and OA treatment.

## Supplementary information

Supplementary Information.

## Data Availability

The datasets generated during and/or analysed during the current study are available from the corresponding author on reasonable request.
