# Supplementary material for: Regulation of Gdf5 expression in joint remodelling, repair and osteoarthritis
Source: Sci Rep. 2020 Jan 13;10:157. doi: 10.1038/s41598-019-57011-8 (PMC6957535; doi:10.1038/s41598-019-57011-8)
Supplement: Supplementary file 1 — Supplementary Information. [file 41598_2019_57011_MOESM1_ESM.pdf]

Regulation of *Gdf5* expression in joint remodelling, repair and osteoarthritis

Karolina Kania[1], Fabio Colella[1], Anna H.K. Riemen[1], Hui Wang[1], Kenneth A. Howard[2], Thomas Aigner[3], Francesco Dell'Accio[4], Terence D. Capellini[5,6], Anke J. Roelofs[1]*, Cosimo De Bari[1]*

[1] Arthritis and Regenerative Medicine Laboratory, Aberdeen Centre for Arthritis and Musculoskeletal Health, University of Aberdeen, Aberdeen, UK

[2] Interdisciplinary Nanoscience Center (iNANO), Department of Molecular Biology and Genetics, Aarhus University, Aarhus, Denmark

[3] Department of Pathology and Molecular Pathology, Medical Center Coburg, Coburg, Germany

[4] Centre for Experimental Medicine and Rheumatology, William Harvey Research Institute, Barts and the London School of Medicine and Dentistry, Queen Mary University of London, London, UK

[5] Department of Human Evolutionary Biology, Harvard University, Cambridge, Massachusetts, USA

[6] Broad Institute of Harvard and MIT, Cambridge, Massachusetts, USA

*Equal author contribution.

Correspondence: Cosimo De Bari, MD PhD FRCP, Institute of Medical Sciences, University of Aberdeen, Foresterhill, Aberdeen AB25 2ZD, UK. Tel: +44-1224-437477, E-mail: c.debari@abdn.ac.uk.

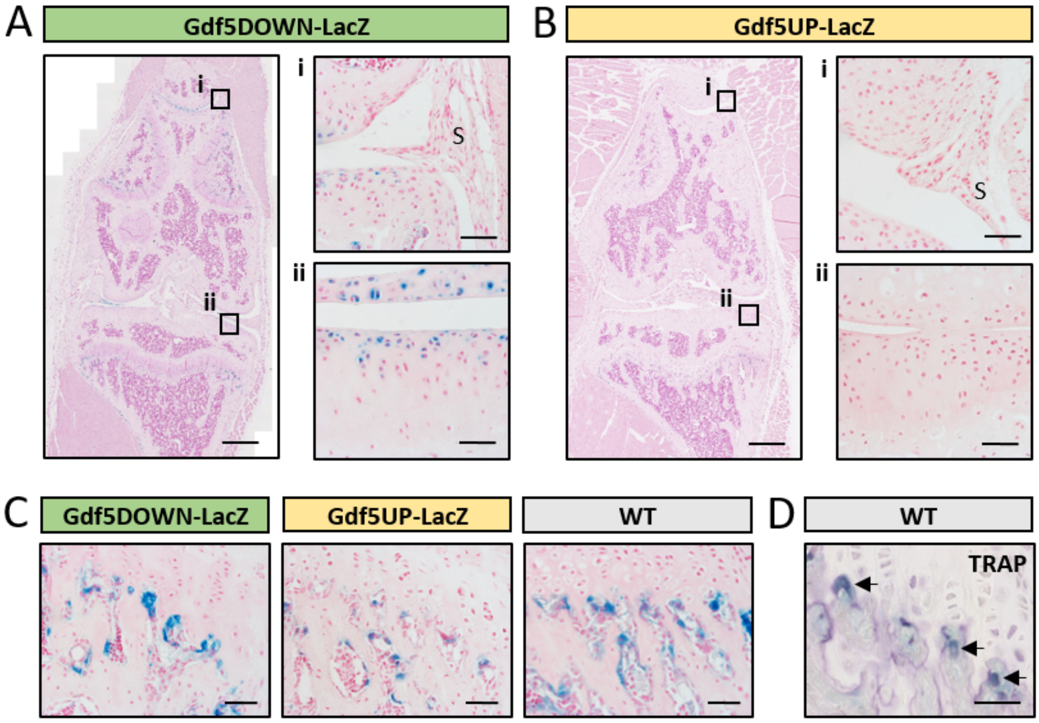

Supplementary Figure 1. *Gdf5* expression in adult knee joint. (A, B) Representative sections from whole-mount LacZ stained knee joints, counterstained with Nuclear Fast Red, of (A) *Gdf5DOWN-LacZ* (n = 10) and (B) *Gdf5UP-LacZ* mice (n = 8). Note absence of staining in synovial (S) tissue (Ai, Bi), and *LacZ*-expressing chondrocytes in articular cartilage in *Gdf5DOWN-LacZ* (Aii) but not *Gdf5UP-LacZ* mice (Bii). (C) Staining underneath the growth plate in both transgenic strains as well as wild-type (WT) mice (n = 5), likely reflecting staining of osteoclasts due to high endogenous beta-galactosidase activity, as previously reported [1]. (D) TRAP staining of osteoclasts (purple stain) showed co-localisation with blue X-gal staining (arrows) in a WT mouse knee just below the growth plate. Scale bars: 500 µm (A, B); 50 µm (Ai, Aii, Bi, Bii, C, D).

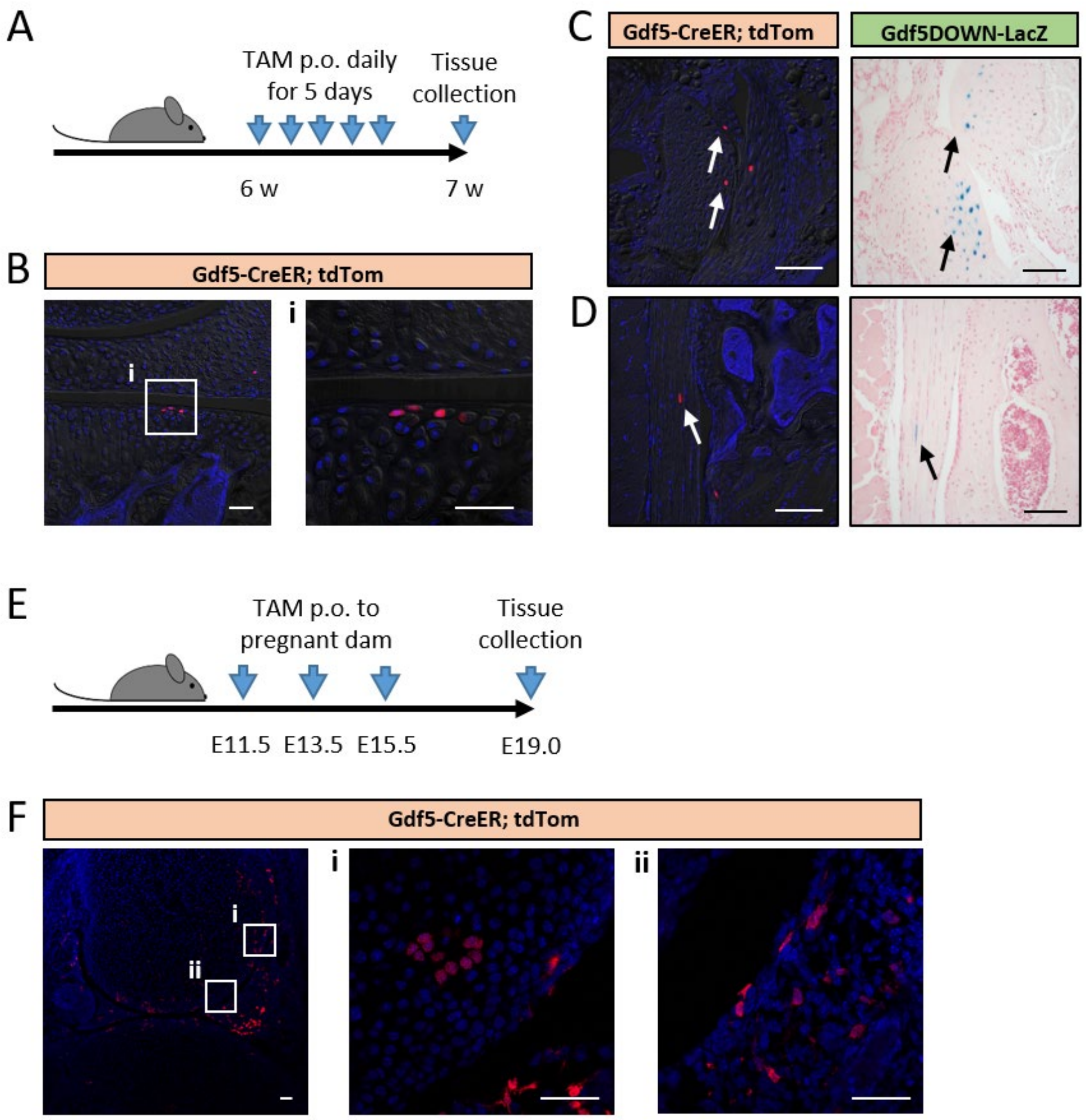

Supplementary Figure 2. *LacZ* expression pattern in *Gdf5DOWN-LacZ* mice reflects activity of the endogenous *Gdf5* promoter. (A-D) Activity of the endogenous *Gdf5* promoter detected in knees of adult *Gdf5-CreER*;*tdTom* mice. (A) Experimental design. (B) Tom expression by cells in the articular cartilage of the tibial plateau detected 2 days after tamoxifen (TAM) administration (180 mg/kg for 5 days) to *Gdf5-CreER;tdTom* mice (n = 2). Scale bars, 50 µm. (C,D) *LacZ* expression pattern in knees from *Gdf5DOWN-LacZ* mice resembles tdTom expression pattern in *Gdf5-CreER;tdTom* mice, showing expression in (C) the cruciate ligament and (D) lateral collateral ligament. Scale bars, 100 µm. (E,F) Activity of the endogenous *Gdf5* promoter detected in *Gdf5-CreER;tdTom* embryos. (E) Experimental design. (F) TdTom+ cells in forming articular cartilage, synovium and meniscus of the developing knee at E19.0 (n = 2), after three doses of tamoxifen administration (120 mg/kg at E11.5 and 160 mg/ml at E13.5 and E15.5) to pregnant dam. Scale bars, 50 µm.

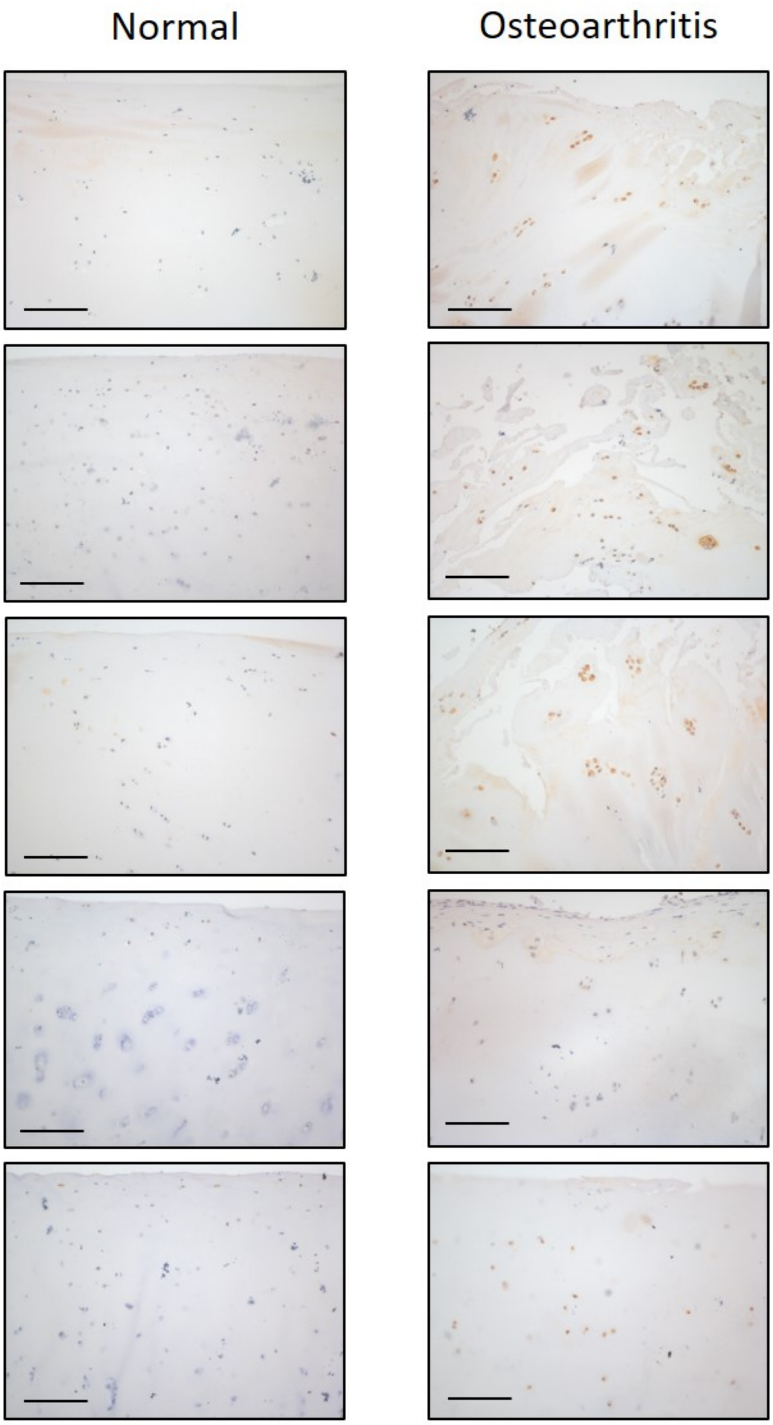

Supplementary Figure 3. GDF5 expression in human articular cartilage. Extended data for Figure 3D. IHC staining for GDF5 in articular cartilage samples from patients with OA (n=5 donors) in comparison to normal cartilage (n=3 donors; 5 joints). Scale bars, 100 µm.

Supplementary Table 1. Antibodies for immunohistochemistry.

Antibody	Clone	Manufacturer	Cat. No.	Conjugation
Col2	polyclonal	Abcam	ab21291	unconjugated
Yap	monoclonal	Cell Signalling Technology	14074	unconjugated
Gdf5	polyclonal	Abcam	ab93855	unconjugated
Goat anti-rabbit IgG	polyclonal	Vector Labs	BA-1000	biotinylated

Supplementary Table 2. DsiRNA sequences for gene knockdown.

Gene	Primer	Sequence (5' to 3')
Yap	Sense	rArUrCrUrUrCrUrGrGrUrCrArArArGrArUrArCrUrUrCrUTA
	Antisense	rUrArArGrArArGrUrArUrCrUrUrUrGrArCrCrArGrArArGrArUrGrU
Taz	Sense	rGrArUrArCrUrUrCrCrUrUrArArUrCrArCrArUrArGrArGAA
	Antisense	rUrUrCrUrCrUrArUrGrUrGrArUrUrArArGrGrArArGrUrArUrCrUrC
Mismatch	Sense	rCrArUrArUrUrGrCrGrCrGrUrArUrArGrUrCrGrCrGrUrUAG
	Antisense	rCrUrArArCrGrCrGrArCrUrArUrArCrGrCrGrCrArArUrArUrGrGrU

Supplementary Table 3. Primers for qRT-PCR.

Gene	Primer	Sequence (5' to 3')
Gdf5	Forward	GCTTTATTGACAAAGGGCAAGA
	Reverse	GGCACTGATGTCAAACACGTA
Yap	Forward	GCCCGACTCCTTCTTCAAG
	Reverse	GAGTGAGCTCGAACATGCT
Taz	Forward	AGCTCAGATCCTTTCCTCAATG
	Reverse	ACCTGTATCCATCTCGTCCAT
Wnt9A	Forward	CAACCTCGTGGGTGTGAAG
	Reverse	CCTCGTGGAAGGGTGCTA
Hprt1	Forward	CAAACTTTGCTTTCCCTGGT
	Reverse	CAACAAAGTCTGGCCTGTATC

Supplementary Reference

1. P. R. Odgren, C. A. MacKay, A. Mason-Savas, M. Yang, G. Mailhot, and M. J. Birnbaum, "False-Positive β-Galactosidase Staining in Osteoclasts by Endogenous Enzyme: Studies in Neonatal and Month-Old Wild-Type Mice," Connect. Tissue Res., vol. 47, no. 4, pp. 229–234, 2006.
